# Principles, practices and knowledge of clinicians when assessing febrile children: a qualitative study in Kenya

**DOI:** 10.1186/s12936-017-2021-7

**Published:** 2017-09-20

**Authors:** Anneka M. Hooft, Kelsey Ripp, Bryson Ndenga, Francis Mutuku, David Vu, Kimberly Baltzell, Linnet N. Masese, John Vulule, Dunstan Mukoko, A. Desiree LaBeaud

**Affiliations:** 1 UCSF Benioff Children’s Hospital Oakland, 747 52nd St, Oakland, CA 94609 USA; 2 Department of Medicine, Hospital of the University of Pennsylvania, 3400 Spruce St, Philadelphia, PA 19104 USA; 3 Department of Pediatrics, Children’s Hospital of Philadelphia, 3400 Spruce St, Philadelphia, PA 19104 USA; 4 Kenya Medical Research Institute, P.O. Box 1578, Kisumu, 40100 Kenya; 5 Department of Environment and Health Sciences, Technical University of Mombasa, P.O. Box 90420 - 80100 G.P.O, Mombasa, Kenya; 6 Stanford University, 300 Pasteur Drive, G312C, Stanford, CA 94305-5208 USA; 7 University of Washington, HMC Box 359909, 325 9th Avenue, Seattle, WA 98104-2499 USA; 8 Vector-Borne Diseases Unit, P. O. Box 20750 – 00202, Nairobi, Kenya; 9 UCSF School of Nursing Center for Global Health, Center for Global Health, Box 0606, San Francisco, CA 94143-0602 USA

**Keywords:** Fever, Malaria, Children, Kenya, Knowledge, Principles, Behavior, Practices

## Abstract

**Background:**

Clinicians in low resource settings in malaria endemic regions face many challenges in diagnosing and treating febrile illnesses in children. Given the change in WHO guidelines in 2010 that recommend malaria testing prior to treatment, clinicians are now required to expand the differential when malaria testing is negative. Prior studies have indicated that resource availability, need for additional training in differentiating non-malarial illnesses, and lack of understanding within the community of when to seek care play a role in effective diagnosis and treatment. The objective of this study was to examine the various factors that influence clinician behavior in diagnosing and managing children presenting with fever to health centres in Kenya.

**Methods:**

A total of 20 clinicians (2 paediatricians, 1 medical officer, 2 nurses, and 15 clinical officers) were interviewed, working at 5 different government-sponsored public clinic sites in two areas of Kenya where malaria is prevalent. Clinicians were interviewed one-on-one using a structured interview technique. Interviews were then analysed qualitatively for themes.

**Results:**

The following five themes were identified: (1) Strong familiarity with diagnosis of malaria and testing for malaria; (2) Clinician concerns about community understanding of febrile illness, use of traditional medicine, delay in seeking care, and compliance; (3) Reliance on clinical guidelines, history, and physical examination to diagnose febrile illness and recognize danger signs; (4) Clinician discomfort with diagnosis of primary viral illness leading to increased use of empiric antibiotics; and (5) Lack of resources including diagnostic testing, necessary medications, and training modalities contributes to the difficulty clinicians face in assessing and treating febrile illness in children. These themes persisted across all sites, despite variation in levels of medical care. Within these themes, clinicians consistently expressed a need for reliable basic testing, especially haemograms and bacterial cultures. Clinicians discussed the use of counseling and education to improve community understanding of febrile illness in order to decrease preventable deaths in children.

**Conclusion:**

Results of this study suggest that since malarial testing has become more widespread, clinicians working in resource-poor environments still face difficulty when evaluating a child with fever, especially when malaria testing is negative. Improving access to additional diagnostics, continuing medical education, and ongoing evaluation and revision of clinical guidelines may lead to more consistent management of febrile illness by providers, and may potentially decrease prescription of unnecessary antibiotics. Additional interventions at the community level may also have an important role in managing febrile illness, however, more studies are needed to identify targets for intervention at both the clinic and community levels.

**Electronic supplementary material:**

The online version of this article (doi:10.1186/s12936-017-2021-7) contains supplementary material, which is available to authorized users.

## Background

Previously, World Health Organization (WHO) guidelines recommended anti-malarials for any child presenting with fever given the high prevalence of malaria as benefit of potential over-treatment outweighed risk. Recently, however, there has been a push toward laboratory-confirmed diagnostics and evaluation for other diseases if malarial testing is negative, given the global decrease in fever caused by malaria [1, 2]. Because the incidence of fevers due to malaria is shrinking, the diagnostic process for children with febrile illness is even more challenging. Clinicians face emerging new diseases in addition to atypical presentations of common childhood infections such as diarrhoeal illness, pneumonia, influenza, and meningitis. In particular many aetiologies may be difficult to diagnose in resource-poor settings given the lack of imaging equipment and laboratory testing [3–5].

Kenya’s Ministry of Health follows the WHO guidelines and government-funded hospitals and clinics now routinely test for malaria in all children who present with fever [6–8]. Outside of the neonatal period, common febrile illnesses including malaria, diarrhoea, and pneumonia are still the leading causes of mortality in Kenyan children [9]. Prior qualitative studies of both clinicians and caregivers in various communities in African countries have indicated that resource availability, need for additional training in differentiating non-malarial viral and bacterial illnesses, and lack of understanding within the community of when to seek care play a role in effective diagnosis and treatment [10–13]. In one prior study, health workers in Zanzibar identified a need for improved point-of-care testing for diseases other than malaria. There was some difficulty in assessing which illness required antibiotics for treatment and which did not. Most Kenyan health facilities only have the capacity for microscopy-aided diagnostics, with no funding available to buy point-of-care tests [14].

Since the WHO recommendations on febrile illness have been updated, studying the principles, knowledge, and practices of health workers in Kenya is extremely important to understand how these changes are being implemented and what barriers stand in the way of providing adequate health care for children. The Ministry of Health in Kenya has promoted mandatory testing for malaria for all children presenting with fever but now, given that health workers must differentiate febrile illnesses in the face of negative malarial testing, there is a need to study how these changes have affected their perceptions and management.

## Methods

Semi-structured key informant interviews were used as previously described [5]. Individual interviews lasting approximately 60 min each were conducted in the health facilities during heath workers’ free time by one member of the study team (AH). Health workers were chosen from three sites in western Kenya: Obama Children’s Hospital, Chulaimbo Sub-district Hospital, and Mbaka Oromo Dispensary all in the Kisumu, Kenya area; and two coastal sites: Diani Health Centre in Ukunda, Kenya and Msambweni District Hospital in Msambweni, Kenya. Obama is a large children’s referral hospital in an urban setting, where Chulaimbo is a district hospital in a rural setting. Mbaka Oromo is a small dispensary in a rural setting. Msambweni is a district hospital in a rural setting and Diani Health Centre is a clinic in an urban setting. Interviews were conducted in English (as all Kenyan health workers use English as their language for medical communication), recorded, and transcribed. The interviews were based off of those used by Baltzell et al. [5] with some modifications to account for differences in resource availability and further explore topics identified by that study (Additional file 1). The Kenyan facilities all had either malaria rapid diagnostic test (RDT) or malaria blood smears readily available. The interview questions were also augmented to include more detail on viral and bacterial illnesses and their management. The study participants were all working in health facilities where externally-funded research studies on malaria and/or arboviral diseases were being conducted. The health facilities used to recruit study participants in this study house a larger arbovirus study on dengue and chikungunya that already obtains clinical data on every febrile child who seeks medical care. This ongoing study tracks children presenting with non-specific febrile illness in urban and rural areas both at the coast and western Kenya sites. Interview questions included the following: work experience and school background, methods and resources for diagnosing febrile illness, and clinician perceptions on treating children and their experiences with this.

### Ethical considerations

This study was approved by the Stanford University IRB (34649), the Children’s Hospital Oakland Research Institute IRB (015-050), and the Kenya Medical Research Institute (KEMRI) Scientific and Ethical Review Unit (015/3104). Key informants provided written informed consent to have information shared during the interview process used in this analysis. Any information potentially used to identify the participants was omitted from recordings and transcriptions.

### Participants

At each study site, participants were identified with the help of local staff and study team members. Participants were chosen based on availability and willingness to participate. Participants were interviewed privately, one at a time, in order to decrease any interference with clinical duties and maintain privacy. Participants did not receive any incentive for their participation. It is important to note that six of the participants (all clinical officers) are part of a larger arboviral disease study in addition to their usual duties evaluating febrile children. In addition, several of the other clinicians are aware of this study as it is concurrently taking place at all of the study sites.

### Analysis

Interviews were conducted in English, audio-recorded and transcribed verbatim by a member of the study team (AH). Clinicians were assigned a number 1–20 to protect their identity. Interviews transcripts were then coded to look for categories and major themes by two researchers (AH and KR) independently using immersion/crystallization style of analysis [15]. Any differences in interpretation were resolved by discussion between the researchers, further review of the transcripts, and clarification with the interviewer until a consensus was reached. Data were analysed for themes characterizing various factors that play a role in diagnosis and management of febrile children in these facilities. The themes identified were then reviewed by two additional members of the study team, one of which is a Kenyan researcher familiar with cultural norms (DV and LM). Participants were assigned a number from 1 to 20 post-analysis and these numbers are included with quotations in “Results” section to allow for consistent tracking and subthemes among the specific providers while maintaining anonymity.

## Results

Twenty health workers in Kenya were interviewed about the evaluation and treatment of children presenting with febrile illness: two nurses, fifteen clinical officers, two paediatricians, and one medical officer. There were a total of ten women and ten men. Clinicians had varying levels of experience and training. Participating health workers were employed by five government-funded clinic sites with ten health workers from the western sites and ten health workers from the coastal sites (Table 1). Each site had standard equipment available including thermometers, stethoscopes, scales, otoscopes and the ability to perform either blood smear or rapid diagnostic tests (RDTs) for malaria on site at the medical facility.

**Table 1:** Clinician details

	Clinician total	Clinicians per site	Male	Title	Arbovirus project	Specific training	
						Malaria	Paediatrics
Site							
West	10	Mbaka Oromo: 3	7	7 CO	2	9	5
		Chulaimbo: 2		2 RN			
		Obama: 5		1 MD			
Coast	10	Msambweni: 6	3	8 CO	4	8	5
		Ukunda: 4		2 MD			

Total number of clinicians per site, number of male clinicians, title (level of training), number of participants per site involved in the arbovirus project, and number who had additional specific training in either malaria or paediatrics

*CO* clinical officer, *RN* registered nurse, *MD* physician

Five major themes were identified:

1. Strong familiarity with diagnosis of malaria and testing for malaria
2. Clinician concerns about community understanding of febrile illness, use of traditional medicine, delay in seeking care, and compliance
3. Reliance on clinical guidelines, history, and physical exam to diagnose febrile illness and recognize danger signs
4. Clinician discomfort with diagnosis of primary viral illness leading to increased use of empiric antibiotics

Lack of resources including diagnostic testing, necessary medications, and training modalities contributes to the difficulty clinicians face in assessing and treating febrile illness in children.

### Theme 1: strong familiarity with diagnosis of malaria and testing for malaria

Clinicians relayed the importance of obtaining an RDT or blood smear when presented with a febrile child and were quite familiar with the current guidelines to test all patients before administering anti-malarials. All facilities had one or both of these testing modalities available and endorsed good adherence to the country-wide clinical guidelines that are in conjunction with WHO policy. Both those who were recently trained and clinicians with more experience were aware of the change in guidelines and had updated training.

*“So long as a child has had fever in this area, you want to do a malaria blood film. Even if they have other symptoms, even if the throat looks very bad, you still want to do, to be sure that they don’t have malaria, because malaria is what will most likely kill them.” (14)*

*“Since we are in a malaria endemic area, all patients who present with fever must be done a BS (blood smear), malaria parasites.” (7)*

Clinicians were aware that malaria is very common, but knew, however, that this can sometimes lead to errors in diagnosis. There was concern for overreliance on a positive malaria test in the context of other signs and symptoms masking a concomitant infection. Many reported difficulty when patients presented after having been previously evaluated at another health centre. Remote facilities were perceived to have an increased tendency to treat malaria regardless of test results, or treat malaria without evaluating the patient further for other conditions.

*“And then, I’ve talked about making a wrong diagnosis. Maybe the child has malaria co*-*existing with a meningitis and you take a child for malaria, malaria comes out positive and you start a child on treatment, forgetting about the meningitis.” (5)*

*“We are overemphasizing malaria a lot. So if a child comes with fever, most of the clinicians or nurses or doctors, they think of malaria first. They don’t think mostly of viral, upper respiratory infection or viral infection or any other.” (15)*

Specific malaria training post-graduation was reported by all but three participants. These three participants had all recently completed their medical training. One of the more experienced clinicians was even trained as a malaria workshop provider. Malaria training was the most commonly reported when asked about additional training provided after graduation. Almost all of these trainings were government sponsored, though one clinician reported having some training provided by a malaria-focused research project. The majority of clinicians, especially those raised in the areas studied (where malaria is endemic) also gave history of a personal experience with malaria. Several had suffered from malaria themselves, some on multiple occasions, and many had family members, neighbors, or friends who had suffered from malaria. All endorsed familiarity with seasonal variation. Many of them said their neighbours and friends often approach them directly when ill with fever. Most said they would then advise these people to go to a local health centre for testing instead of providing medication to them personally. The clinicians themselves said they also went to health facilities for testing rather than self-medicating.

*“Yes I’ve had trainings on case management, malaria case management where we talk about first line management for uncomplicated malaria, second line treatment, and* etc*. for even complicated malaria too.” (18)*

*“…it is rare to grow in this region without an attack of malaria. All of my family members have suffered.” (3)*

### Theme 2: clinician concerns about community understanding of febrile illness, use of traditional medicine, delay in seeking care, and compliance

Despite familiarity and acceptance of routine testing for malaria and a decrease in overall prevalence of malaria in Kenya, many clinicians said they still see critically ill children on a regular basis. Several felt this could be due to delay in seeking care and lack of recognition of fever or danger signs by caregivers. They think many caregivers would prefer to consult first with a traditional healer. They also feel patients have difficulty with access to care, perform self-medication, or even have courses of malaria treatment prescribed by outside clinicians without testing.

*“…but they take too long at home that by the time they arrive at the facility or when they are almost arriving at the health clinic it is too late, not much can be done.” (9)*

*“Most of them… …they prefer giving their own treatments at home and when there is no improvement is when they access the health care worker for management, mainly.” (20)*

Despite public health initiatives by the government and non-governmental organizations (NGOs), including mosquito net distribution, clinicians reported that there were still challenges with vector control and adherence to public health and prevention measures at the community level.

*“Ok there is still not enough coverage of mosquito nets. Even though the government did a mass campaign of distributing the net.” (18)*

Clinicians, especially those in more rural facilities, endorsed a need for better education at the community level. Clinicians felt there was decreased health awareness and increased risk for developing infectious diseases in areas with lower socioeconomic status and levels of education. They gave examples of ways to improve community awareness, including implementing outreach measures and mobile education modules similar to what some said are already being done by public health workers in villages for other health topics, such as women’s health, immunizations, and family planning. Some clinicians felt that providing counseling and education at the individual level was an important part of their role.

*“I think we need to have some health education to be given to those caretakers at home. We conduct outreach, health talks at the hospital, so they can know what is supposed to be done when a patient is having a fever and who exactly is supposed to be taken to the hospital.” (12)*

*“Because you find they do not have proper hygiene methods, or the infrastructure is poor, so you find there is a lot of water pooling in different areas, there is the bushes, the mosquitoes are also there.” (16, discussing the villages where children come frequently with malaria)*

### Theme 3: reliance on clinical guidelines, history, and physical exam to diagnose febrile illness and recognize danger signs

All clinicians, regardless of experience or location, relied on their history taking and clinical skills given lab tests were often unavailable or unreliable. In the febrile child, clinicians would first evaluate for any danger signs and then continue to evaluate further based on the details of each case.

*“Typical for malaria three things: history, clinical exam and third is lab.” (3)*

*“Take the temperature first, you take a history, take a full history about a child, where do they stay, what are they doing, whether they presented at another hospital, are they taking that? Do a physical examination from head to toe.” (15)*

Clinicians commonly mentioned the use of clinical guidelines including the government-distributed Paediatric Protocol and the WHO’s Integrated Management of Childhood Illnesses (IMCI). Clinicians with training or working in paediatric-focused health centres reported carrying the Paediatric Protocol (a small paper booklet) with them at all times and referring to it when confronted with an unfamiliar problem.

“*We have guidelines for malaria yes. Specifically for paediatrics, no. But we have some guidelines for infections that we were taught. We have the IMCI book, it is this one. I have it and it helps in diagnosing the infections in young children. Normally I use them. Normally we refer, if we need a test and we are not sure which, we are referring to make a good classification.” (2)*

*“Yeah we have our paediatric protocol and that is the most important tool that we have… yeah we use it a lot, it is very, very, helpful.” (20)*

When a child’s symptoms or test results did not clearly fit the guidelines, clinicians were less confident in their diagnosis and management. This varied based on training and experience, with more specialized clinicians (including those clinical officers who had specialized in paediatrics) and those working in a paediatric health centre being more comfortable diagnosing and evaluating children. These clinicians, however, also typically had more access to additional laboratory evaluation or the ability to refer the child to other laboratory facilities for further testing.

*“We are using antibiotics so much and according to the IMCI guidelines, when there is fever and there is no other positive cause of fever that you can think of, the IMCI does not cover the throat infection, and that guideline.” (13)*

*“It is easier for me to make a diagnosis in a child than someone who has not worked in a children’s hospital.” (19)*

### Theme 4: clinician discomfort with diagnosis of primary viral illness leading to increased use of empiric antibiotics

All clinicians had received teaching on the differences between viral versus bacterial illness early in training and when to use antibiotics. The majority of clinicians understood that viral illness are self-limiting and do not respond to antibiotics. There was some variation in the understanding of how a bacterial versus a viral illness might present, including the symptoms, acuity of illness, duration, incidence, and severity. Many clinicians believed that bacterial illnesses are much more common than viral illnesses in febrile children.

*“…a bacterial illness, can be treated with antibiotics whereas a viral illness cannot be treated, just self*-*limiting*.” (*11)*

*“So predominantly we have more bacterial infections as opposed to viral infections.” (16)*

Although clinicians had some understanding of pharmacology and microbiology in relation to viral and bacterial diseases, there were many other factors that influenced the prescription of antibiotics. Reasons given for prescribing antibiotics included: pressure from families, discomfort with the possibility that the child could worsen before returning to care, the perception that bacterial illnesses are much more common, understaffing, and inability to differentiate causes of illness based on history and physical examination alone. The degree of reported antibiotic use ranged from clinicians who said they would prescribe antibiotics for anything that was not malaria, to others who would try to reserve them only for cases that they truly thought had a bacterial infection.

*“So they will come back they have a very severe illness. So it is like we are giving the antibiotic for prophylactic purposes because you don’t want the child to complicate.” (12)*

*“Sometimes in other cases, you find it is cold, you just have to give self*-*remedy but the mother insists, ‘You have just given me piriton antihistamine and paracetamol and it’s febrile illness, why have you just given me paracetamol?’” (6)*

More experienced or more highly trained clinicians expressed greater comfort with not prescribing antibiotics when they diagnose a viral illness. They were more comfortable watching and waiting if the patient was relatively well-appearing.

*“Yeah, if we think this is most likely viral infection than I am uncomfortable to prescribe antibiotics because you know if you give that antibiotic it will not help much.” (2)*

*“So true, a number of times if I feel this is a viral illness I won’t give them an antibacterial, I will just monitor them. So long as you can monitor them closely, you are fine.” (14)*

### Theme 5: lack of resources including diagnostic testing, necessary medications, and training modalities contributes to the difficulty clinicians face in assessing and treating febrile illness in children

All of the clinicians included in this study were providing clinical care for paediatric patients on a regular basis, and most were working primarily in paediatrics. Despite this, only 7 of the 20 reported having any specific additional training or specialization in paediatrics (with one other reporting only training in neonatal resuscitation), with 6/7 of these clinicians working in the same paediatric institution. Most of those who had specialty training had pursued this on their own. Clinicians at all of the sites said continuing medical education (CME) was provided and available in their area, but many could not access it due to time constraints, distance, and understaffing. Clinicians at the larger, central facilities said it was easier to attend government-sponsored training.

*“No, but here they do the training on IMCI, and I have not done IMCI yet.” (19)*

*“We try to have it [CME] weekly, but it depends on how bad or how good the ward is. So when we have critical patients it becomes difficult.” (16)*

Among study sites there was variation in laboratory capacity with the most equipped paediatric hospital able to perform basic tests and refer patients to the main hospital laboratory for more specialized testing while smaller clinics were limited to only an RDT.

Several clinicians had worked in various facilities during their careers ranging from private clinics, to NGOs, to central and peripheral government facilities. They acknowledged significant differences in the availability of resources and need to improvise depending on where they were working. Many indicated that private clinics had many more resources and abilities to provide better care because of their funding. As all were currently working in government facilities, this provided many challenges in feeling confident in how they were treating and managing children with fever.

*“But when I am working with the government, I realize that it is hard. Yeah it is hard. Because they don’t do a lot of investigations.” (4)*

*“If you work at the general hospital, most of the time you have the bare minimum. If you go to the private hospital you can get anything you want.” (14)*

Even at the larger, better-equipped hospitals clinicians were frustrated that laboratory equipment was often broken, unreliable, too slow, or lacked the proper supplies for effective use. In the smaller clinics, clinicians also had problems with obtaining basic tools, such as thermometers and stethoscopes, to do a complete physical examination, and said it often made their job more difficult as they were sharing tools among departments. Clinicians felt that a full haemogram would be the most useful test to help them in determining the cause of fever when malaria testing is negative. Several indicated the white blood cell count is useful in differentiating between bacterial and viral illness.

*“We need things like, like right now in that IMCI clinic, we only have one thermometer. It is constantly moving from here to there in casualty. So that is frustrating. We do not have enough equipment to diagnose fever.” (10)*

*“Hemogram is very useful, and then the blood slide. Those are the main tests. Cause they give us, in fact, they help us make most of the diagnosis.” (20)*

Besides laboratory testing and basic equipment, lack of personnel was also brought up. Clinicians felt this distracted from their ability to provide adequate clinical care and also attend trainings and improve their clinical skills.

*“And then sometimes, the workload compared to the number of staff that we have, like it reaches a time where you can’t think anymore. And it is difficult to really to think and the patients, your mind needs to be like, clear.” (10)*

*“We have really a shortage of staff. There’s a problem.” (20)*

Finally, even when clinicians were able to make a diagnosis or narrow down the differential, they often did not have the first line or preferred treatment, or were unable to perform a necessary procedure. All reported stock outs on various occasions, more commonly in smaller clinics. Clinicians were usually forced to refer these patients to either buy medications or try and obtain them at another clinic. Many would provide an alternative even though they knew it would not be effective.

*“But sometimes we send those patients they want to go to a bigger hospital to do the procedure but normally I improvise to do anything which can help.” (6)*

*“At times, like I said before, you use them according to the guidelines, but at times you don’t have those drugs according to the guideline so you are forced to do what can really help the client.” (18)*

## Discussion

Kenyan clinicians in malaria endemic zones have many factors influencing how they manage a child presenting with fever in the age of mandatory malaria testing. Many of the themes identified were consistent with those reported in health workers in Zanzibar and other studies in health workers in East Africa [3, 4, 10, 11]. This study was able to expand on this by interviewing clinicians who work primarily with children in a variety of clinical settings in areas where malaria prevalence is overall decreasing, but still quite high [1, LaBeaud, pers. comm.]. The similarity of themes identified in Kenyan clinicians with those in other regions, despite some variations in their government healthcare systems, further highlights that these data are highly relevant and generalizable for areas of sub-Saharan Africa. This is especially true in malaria-endemic areas as changes in the WHO guidelines are forcing clinicians to stop treating empirically with anti-malarials and evaluate children for other causes of fever when malaria testing is negative. This becomes increasingly important as rates of malaria transmission decrease.

Clinician familiarity and reported adherence to malaria testing guidelines is a testament to the efforts made by governments and NGOs in improving malaria evaluation and treatment. All but three clinicians reported having updated training specific to malaria beyond that which was provided during their schooling. Those who had not had more specific training had only recently completed their schooling. Regardless of level of training/familiarity with malaria, clinicians felt very limited in what they could do for a patient once a malaria test was negative. Even those who felt more comfortable making a diagnosis or treating empirically for the next likely illness on their differential were still limited by lack of available resources, and often resorted to prescribing a treatment they knew would not be effective. Difficulty with resource limitations was consistent with that seen in other studies in various parts of Africa, including some with limitations in their ability to provide malaria testing, which was not an issue in this study [3, 10, 11].

This study further characterizes some of the beliefs and behaviours associated with viral versus bacterial illness and antibiotic prescription. Similar to clinicians in Zanzibar, many Kenyan clinicians said they prescribed antibiotics when they were not sure of the diagnosis or even when they thought bacterial illness was unlikely. Clinicians gave various differentials for fever including upper respiratory infection, pneumonia, urinary tract infections, meningitis, sepsis, otitis media, and viral illness (including dengue and chikungunya, the diseases in the arbovirus study), among others. Clinicians with more experience and training were more likely to mention common paediatric illnesses in their differentials and describe the methods they used to diagnose them. Even those who seemed to have a good conceptualization of some of the differences between bacterial and viral illness would still prescribe antibiotics in likely viral illnesses because of fear the child would worsen, their inability to ensure adequate follow up, and pressure by caregivers to provide medication. Their fears are understandable given the limitations of working in facilities with minimal laboratory resources. Despite having access to RDTs, providers still have no consistent way to distinguish viral versus bacterial illness other than their clinical acumen. In a prior study, patients with an available RDT result had higher percentages of antibiotic prescription [16]. Given this, it is very likely that perception of the number of febrile children with bacterial infections is likely overestimated, however, leading to over-prescription of antibiotics. As seen in previous studies in Tanzania and Uganda, it is plausible that a significant portion of children presenting with fevers may truly have bacterial infections, however, these are still a minority of cases [17–24].

The majority of clinicians interviewed had not had specific training in paediatrics despite that all of these clinicians see a high percentage of paediatric patients and are supposed to be using IMCI guidelines. The amount of training and clinician confidence levels were variable regarding topics in paediatric health and febrile illness, especially in the larger urban paediatric hospital (Obama Children’s). Those who had more paediatric training, especially the paediatricians, described being more comfortable watching and waiting if a child presenting with a fever and negative malaria testing was clinically stable.

The correlation of increased level of training or experience with increased comfort in observing a febrile child with a likely viral illness makes a case for strengthening paediatric training and familiarity with guidelines such as the IMCI. Prior studies have shown that adherence to IMCI guidelines by clinicians in low-resource settings is poor, though it also not proven that implementation of these guidelines actually impacts mortality [25–28]. These guidelines may often also be used in settings, as in this study, where clinicians have not been adequately trained in how to use them. A recent study in Tanzania found that clinicians reported several other factors besides a lack of training contributed to IMCI adherence including high workload/patient volume and a lack of belief in their importance [29]. Clinicians in this study also expressed discomfort when dealing with conditions that deviated from the IMCI flow chart. Collectively these data suggest that ongoing revision of IMCI is necessary along with further studies to prove their efficacy. In addition, as several providers stressed the importance of the history and physical examination in their decision-making, providing further training in clinical reasoning and critical thinking may also lead to improved recognition of less common illnesses and facilitate proper referral and escalation of care within the healthcare system.

Public health factors and caregiver understanding were substantial barriers that clinicians felt prevented them from providing adequate care. Though clinicians were very proficient in following guidelines for testing and treatment of malaria, there was obvious frustration with caregivers who brought in children who were already very ill because of delay in seeking care. This was more evident in the rural facilities, especially in Msambweni. Despite bed net distribution programmes and efforts to reduce standing water near households in Kenya, clinicians felt caregivers still did not understand or comply with these interventions, and often had poor recognition of paediatric fever and its implications for a child [30, 31]. Interviews with caregivers in Tanzania and Malawi found similar themes in their interviews with caregivers [10, 11]. Many caregivers did not understand what a fever was and reported bringing their children to traditional healers prior to or in addition to community health centres. Clinician in this study brought up ideas for improving education in communities and the importance of their role as patient and community educators.

Limitations of this study include that the majority of participants were more familiar with research and potential causes of viral illness in children given their awareness of arboviral diseases and findings; therefore, study findings may not represent clinicians in facilities where no such research takes place. To attempt to reduce this bias, we interviewed participants who were not part of the arboviral study. Participation by six of the clinical officers in a dengue and chikungunya study and that resultant training may have altered their clinical management of children with fever; however, the same themes emerged between those who were or were not part of the arboviral study, and this would be expected to cause more awareness of viral illness and potential causes of fever. This study was limited to only 20 participants in two regions, and therefore, may not be generalizable to the majority of Kenyan clinicians, or other clinicians working in other parts of Africa. In addition, most of the study participants were clinical officers, and although they commonly are the first to evaluate children presenting to a government facility with fever in Kenya, we did not include all others who may have this role such as community health workers, medical assistants, students, interns, or registrars. To further reduce bias, the study interviewer had not previously worked with the study team and was not familiar with any of the clinicians before the study was conducted.

Additional studies are needed to better understand management of febrile children by clinicians in resource-poor settings as the dynamics of malarial illness continue to evolve. Direct examination of clinician behavior and correlation of quantitative data is necessary to further elucidate how clinicians are actually diagnosing and treating children with fever, and if they are truly adhering to the malaria testing and treatment guidelines. Further exploration of antibiotic prescription patterns is also needed. Finally, better needs assessment and public health investigations at the community level are necessary to determine where interventions with the most impact can be made.

## Conclusion

Though malaria is decreasing in Kenya, it is still highly prevalent in endemic areas. Clinicians working in resource-poor environments in these areas are faced with several challenges in evaluating a child with fever, especially when malaria testing is negative. Improved access to education and diagnostics for clinicians may lead to more comfort in diagnosing viral illnesses and potentially decreased prescription of unnecessary antibiotics. Paediatric guidelines and directives for clinicians of all levels also need to be continuously evaluated and improved, especially as access to additional testing increases. Community education and public health interventions may also have an important role in improving provider understanding of fever and appropriate presentation to healthcare facilities. More studies are needed to identify targets for intervention at both the clinic and community levels.

## Additional files

**Additional file 1.** Semi-structured interview guide (modified and reproduced from Baltzell et al. [5] with permission).

**Additional file 2.** Consent for Participation in Study.

## Abbreviations

IMCI: Integrated Management of Childhood Illness
CME: continuing medical education
WHO: World Health Organization
RDT: rapid diagnostic test
