# Supplementary material for: Principles, practices and knowledge of clinicians when assessing febrile children: a qualitative study in Kenya
Source: Malar J. 2017 Sep 20;16:381. doi: 10.1186/s12936-017-2021-7 (PMC5607512; doi:10.1186/s12936-017-2021-7)
Supplement: Supplementary file 1 — Additional file 1. Semi-structured interview guide (modified and reproduced from Baltzell et al. [5] with permission). [file 12936_2017_2021_MOESM1_ESM.docx]

Additional file 1: Semi-structured interview guide (modified and reproduced from Baltzell et al. 2013 with permission)

Study participant ID #____________________

Date of interview ________________________

Length of interview in minutes ______________

1. *Background and training*

**Concept: How have diagnostic skills changed over time and what were influences that resulted in change**

Do you live in this district? How long have you worked here? How often do children present to this clinic with fever?

Where did you grow up? How prevalent is malaria there? Other diseases?

Why did you decide to become a (nursing assistant, clinical officer, nurse)?

Have you worked as a clinician anywhere else? (If yes, what was the experience like at that setting?)

Tell me about your own experiences with febrile and malarial illness.

Tell me about your medical training. (dates, schools, area of focus or research)

Since graduating (or ‘in the past 5 years’), have you had any training specific to:

- malaria?
- febrile illnesses?
- paediatric health?
- (if yes, who ran it? When/how often?)

Is there continuing education offered at this clinic, or at the district level?

Tell me about your work day, how many children do you see? What types of illnesses?

1. *Priorities in diagnostic process*

**Concept: What are the most important things/observations that you employ when diagnosing a child with fever?**

What are the most important things to look for when deciding how to diagnose a child who presents to the clinic with a fever? Why?

What symptoms make you the most concerned about this patient? Why?

What makes you think a child has malaria, or not? Why?

What are other potential diagnoses that you look for when you determine that a child with fever does not have malaria?

Follow up: tell me what symptoms a patient has that make you concerned that they have (potential diagnosis)? Why do you associate these symptoms with that diagnosis?

How do you distinguish between different types of (respiratory illness, etc.)

What tools do you use to test for or rule out these diagnoses? Why do you choose these/this tool?

What do you normally prescribe for (potential diagnosis)? Do you have these in stock?

Are there seasons associated with certain diseases? What are they? Where did you learn this information?

Are there any other methods that you use to figure out the cause of a child’s fever? Why or why not?

Please explain your understanding of a viral illness versus a bacterial illness.

Where in your training were you taught which medications should be used to treat an illness?

Are there times when you do not feel comfortable prescribing antibiotics? If yes, when or why?

1. *Diagnostic procedure and tools*

**Concept: What diagnostic tools/procedures are used at this site most frequently and why. What else is needed?**

What diagnostic tools do you have at the clinic to use when you see a child with fever? Which ones do you find most useful? Why?

Are there any handbooks available at the clinic with guidelines on malaria, febrile illnesses, or paediatric health? Do you use them? How often?

How is your method of diagnosing different from other health care providers you have worked with?

Do you feel that you have the tools you need at this clinic to determine the cause of a fever in a child without malaria? Why or why not?

III. *Perception of reasons behind continuing febrile illness in children*

**Concept: Why does the health care provider believe so many children are still sick? What is needed in Kenya’s current system to improve care and how will this improve health care delivery and diagnostic accuracy?**

Even though malaria has decreased in this area, why do you think children are still dying from febrile illness?

Are there any other considerations you make in this process?

Anything else we didn’t ask?
