# Supplementary material for: Principles, practices and knowledge of clinicians when assessing febrile children: a qualitative study in Kenya
Source: Malar J. 2017 Sep 20;16:381. doi: 10.1186/s12936-017-2021-7 (PMC5607512; doi:10.1186/s12936-017-2021-7)
Supplement: Supplementary file 2 — Additional file 2. Consent for Participation in Study. [file 12936_2017_2021_MOESM2_ESM.doc]

**Consent for Participation in Study**

**DESCRIPTION:** You are invited to participate in **a research study** on evaluation, diagnosis and management of febrile children. The purpose of this study is to determine what factors influence how you evaluate, diagnose, and treat a child who presents to the hospital acutely ill with a fever. You will be asked to participate in a structured interview that will be audio-recorded. These audio recordings will then be de-identified and transcribed and the audio portion will be erased. The transcribed portion will be analyzed to assess for common themes and ideas. This information may be shown at scientific meetings, published in a written format, or used to develop new education modules, diagnostic tools, or treatment protocols.

**TIME INVOLVEMENT:** Your participation will take approximately one hour.

**RISKS AND BENEFITS:** There is a minimal risk that can be associated with your participation in this study in case any information you provide can be directly linked to you. To prevent this from happening, your employers and supervisors will not have any knowledge of who participated in the study or refused to participate. Study ID codes will be used to protect your identity from study directors, your employers, and the general public. There will be no dissemination of any identifying information alluding to who provided which responses and results will be presented without any identifying factors.

The benefits which may reasonably be expected to result from this study are a better understanding of what factors influence how you evaluate, diagnose, and treat children with fever. We will not provide individual instruction or feedback in order to protect your identity. We will, however, use this information for potential development of new education modules, diagnostic tools, and treatment protocols. **We cannot and do not guarantee or promise that you will receive any benefits from this study.** Your decision whether or not to participate in this study will not affect your employment or role in data collection for the current study.

**PAYMENTS:** You will not receive any payment for your participation.

**PARTICIPANT’S RIGHTS:** If you have read this form and have decided to participate in this project, please understand your **participation is voluntary** and you have the **right to withdraw your consent or discontinue participation at any time without penalty or loss of benefits to which you are otherwise entitled**. **The alternative is not to participate.** You have the right to refuse to answer particular questions. The results of this research study may be presented at scientific or professional meetings or published in scientific journals. Your identity or any identifiable data will not be included in these results.

**CONTACT INFORMATION:**

***Questions:*** In case you have any question about the study you can ask it. You can also raise your questions, concerns or complaints about this research, its procedures, risks and benefits, to Dr. Francis Mutuku at +254 (0) 20 2333758 for those in Coastal Kenya; or Dr. Bryson Ndenga +254 (0) 722 932 350 for those in Western Kenya; or the Protocol Director, Dr. Desiree LaBeaud at +1 650-725-6568.

***Independent Contact:***  If you are not satisfied with how this study is being conducted, or if you have any concerns, complaints, or general questions about the research or your rights as a participant, please contact the Kenya Medical Research Institute Scientific & Ethics Review Unit (KEMRI-SERU) Secretary at +254 717 719 477 or +254 202 722 541; or Stanford Institutional Review Board (IRB) to speak to someone independent of the research team at +1 (650)-723-2480 or toll free at 1-866-680-2906. You can also write to the KEMRI-SERU Secretary, P. 0. Box 54840 Code 00200, Nairobi, Kenya; or the Stanford IRB, Stanford University, 3000 El Camino Real, Five Palo Alto Square, 4th Floor, Palo Alto, CA 94306.

I give consent to participate in this study.

___Yes

Indicate ***Yes*** or ***No***:

I give consent to be audiotaped during this study.

___Yes ___No

I give consent for tapes resulting from this study to be used for transcription and analysis of themes and ideas.

___Yes ___No

**The extra copy of this signed and dated consent form is for you to keep.**

**SIGNATURE _____________________________ DATE ____________**
